# An Exploratory Study of Student Pharmacists’ Self-Reported Pain, Management Strategies, Outcomes, and Implications for Pharmacy Education

**DOI:** 10.3390/pharmacy6010011

**Published:** 2018-01-22

**Authors:** David Rhys Axon, Carlos Hernandez, Jeannie Lee, Marion Slack

**Affiliations:** Department of Pharmacy Practice and Science, University of Arizona College of Pharmacy, Tucson, AZ 85721, USA; chernandez@pharmacy.arizona.edu (C.H.); jlee@pharmacy.arizona.edu (J.L.); slack@pharmacy.arizona.edu (M.S.)

**Keywords:** chronic pain, medication use, pain self-management, pharmacy education, student pharmacists

## Abstract

The objective of this study was to describe the prevalence, management strategies, and outcomes of pain experienced by student pharmacists, and to discuss implications for pharmacy education. A questionnaire administered to student pharmacists collected data about their experience, management strategies, and outcomes of pain. Data were analyzed using *t*-tests, chi-square or Fisher’s tests, and logistic regression. Of the 218 student pharmacists who completed the survey, 79% experienced pain in the past five years. Chronic pain impacted students’ ability to work (15%) and attend school (9%). Respondents most commonly used prescription (38%) and over-the-counter (OTC, 78%) non-steroidal anti-inflammatory drugs (NSAIDs), and rest (69%) to manage pain. Men used more opioids, whereas women used more OTC NSAIDs (*p* < 0.05). Emergency department visits were associated with increased prescription drug use to manage pain. This study found that 15% of student pharmacists had chronic pain in the past five years, which was managed with medical and non-medical strategies.

## 1. Introduction

Pain is defined as “an unpleasant sensory and emotional experience associated with actual or potential tissue damage, or described in terms of such damage” [1]. Pain may be acute, defined as “pain that comes on quickly, can be severe, but lasts a relatively short time” or chronic, defined as “ongoing or recurrent pain, lasting beyond the usual course of acute illness or injury or more than three to six months, and which adversely affects the individual’s well-being” [2]. Pain is always subjective [1,3], which can make appropriate diagnosis and treatment difficult.

Pain is prevalent among adults from the United States (US) with an estimated 100 million to 126 million US adults experiencing chronic pain [3,4,5] and is considered the main cause of adult disability in the US [3]. Pain has typically been regarded as a symptom of other medical conditions; however, pain is increasingly being recognized as a disease state in its own right [6]. Pain is a common cause of physician visits, medication use, disability, and leads to poor quality of life [3]. Pain also results in significant economic burden, for both patients and society, and is estimated to cost $560 to $630 billion per year [3]. However, there is a lack of information on the prevalence of pain, strategies used, and outcomes of pain management among college students, in particular pharmacy students. Pharmacy students are of interest since they are trained to understand the mechanisms behind pain medications and should be familiar with the proper use of medications for pain.

Therefore, the objectives of this exploratory study were: (1) to describe the prevalence of pain, pain management strategies, pain outcomes, and factors associated with prescription medication use among this sample of student pharmacists; (2) to assess differences between individuals with acute and chronic pain; and (3) to discuss the implications of these findings for pharmacy education. Based on conventional wisdom, it was hypothesized that the prevalence of pain among this generally healthy, young sample would be low, and that they would utilize pharmacological pain management strategies more often than non-pharmacological methods due to their scientific backgrounds and knowledge about pharmacotherapy.

## 2. Materials and Methods

This cross-sectional descriptive study used data obtained from a paper-based, in-class questionnaire administered to first (*n* = 120), second (*n* = 101) and third-year (*n* = 97) student pharmacists in the US from the classes of 2017 to 2019 in fall 2015. Participants were given information about the study, reminded of their privacy rights, and advised that study participation was strictly voluntary before survey administration began. Students who did not attend class on the day the questionnaire was administered were excluded from the study. The University Institutional Review Board assessed the ethics and approved the study.

The questionnaire was adapted from Breivik et al. [7] and consisted of 24 items across four sections (Supplementary Materials). The first section (five items) asked how often participants experienced pain, and about pain intensity, pain tolerability, and pain location. Along with those who did not experience pain, participants that selected ‘I don’t remember…’ when asked about when they last experienced pain were considered the ‘no pain’ group and were directed to the demographic section. Participants who reported experiencing pain were asked whether the pain was acute (experienced pain occasionally), chronic (experienced pain almost every day for three of the past six months), or chronic intermittent (experienced reoccurring pain with pain free intervals). The second section (seven items) explored participants’ methods to manage their pain, including use of prescription medications, over-the-counter (OTC) products, and non-medical strategies. Participants were also asked about how effective these methods were, how satisfied they were with these strategies, and to report any side effects experienced. The third section (seven items) investigated the outcomes of pain management strategies used by the participants, and the extent to which pain interfered with their daily living. The final section (five items) collected demographic information such as age, gender, and year in pharmacy program.

Data were entered into a Microsoft Excel (2011) spreadsheet according to a codebook for analysis. Respondents were grouped into one of three groups (acute pain, chronic pain, or no pain) for analysis, and then grouped by gender for the comparison between men and women. Due to the low number of respondents, the chronic and chronic intermittent pain categories were merged to form the chronic pain group [8]. Data were compared using independent *t*-tests for continuous variables (pain intensity, level of pain tolerated, pain level using pain management strategies, and number of emergency department (ED) visits) and either a chi-square test or Fisher’s exact test (if the expected value of any one of the cells was below five) for categorical variables. Logistic regression was performed using SPSS (IBM SPSS, v23, Armonk, NY, USA) to identify factors associated with prescription medication use. The dependent variable was ‘use of prescription medicines’, formed by grouping respondents as users of prescription medications if they reported using one or more of the prescription drugs listed, or non-users if they did not report using any prescription medications. Independent variables were determined by performing a univariate analysis of all potential variables. Those that were statistically significant in the univariate analysis were included in the logistic regression model. Independent variables included type of pain (acute or chronic), number of ED visits in the past five years due to pain, and the level of interference pain caused on activities of daily living. The a priori alpha level was set as 0.05.

## 3. Results

A total of 218 student pharmacists completed the questionnaire with a response rate of 69%. The demographic and pain characteristics of study participants were stratified according to the type of pain reported (acute, chronic, or no pain) and are shown in Table 1. More students reported having acute pain (64%) than chronic pain (15%), while 21% of students said they had no pain. Most respondents were aged 19–25 (70%) and women (59%). There were no significant differences in demographic characteristics (age, sex, and year in program) between acute, chronic, and no pain groups (*p* > 0.05). Headache was the most commonly experienced pain by the students (acute 41%; chronic 24%), followed by pain in the abdomen (acute 19%; chronic 15%) and legs/feet (acute 15%; chronic 21%). The back, neck/shoulders, arms/hands, and chest were the least common locations of pain (each reported by less than 12% of respondents). There were no significant differences between acute and chronic pain groups on location of pain. Pain intensity was significantly greater in the chronic pain group (mean = 5.9, SD = 1.7) than in the acute pain group (mean = 5.2, SD = 2.0; *p* = 0.035), whereas the level of pain tolerated did not differ significantly between the chronic pain group (mean = 6.3, SD = 1.8) and acute pain group (mean = 6.1, SD = 1.9; *p* = 0.544).

The strategies used by students to manage their pain, stratified by type of pain and gender, are shown in Table 2. Nearly all participants (99%) used medical strategies and most (96%) used a combination of medical and non-medical strategies to manage their pain. Pain was particularly burdensome for students with chronic pain in terms of the number of pain management strategies used. Students with chronic pain used an average of 8.3 (SD = 4.7) pain management strategies, whereas students with acute pain used an average of 5.6 (SD = 2.9) pain management strategies. When asked to identify the most effective strategy for managing pain, 22% of students identified NSAID use, 19% rest, 17% taking some form of medication, and 10%, acetaminophen use.

The most commonly used medical strategies (see Table 2) were prescription non-steroidal anti-inflammatory drugs (NSAIDs, 38%) and OTC NSAIDs (78%), whilst the most commonly reported non-medical strategy was rest (69%). More participants with chronic pain used medical strategies (prescription NSAIDs, muscle relaxants, physical therapy, transcutaneous electrical nerve stimulator (TENS), steroid injections) and non-medical interventions (herbals or dietary supplements, physical activity, and avoided specific activities) compared to students with acute pain (*p* < 0.05). Opioid use was higher in men than women, whereas women reported using OTC NSAIDs, rest, relaxation or stress reduction techniques, and changing body position more often than men (*p* < 0.05).

The outcomes reported by student pharmacists using pain management strategies are shown in Table 3. Over one-third (37%) of participants were unsatisfied with their pain management strategies. Ten percent of students experienced side effects, and nine percent reported poor or fair health status. The mean level of pain when using pain management strategies was similar between the acute pain group (3.0, SD 2.2) and the chronic pain group (3.2, SD 2.4). Pain interfered ‘quite a bit’ or ‘very much’ with student pharmacists’ ability to: perform daily activities (6%); participate in leisure activities (7%); work (9%); attend school (6%); and have relationships with other people (6%). Among participants who reported that their pain affected their ability to perform daily activities ‘quite a bit’ or ‘very much’, there were significantly more participants with chronic pain (15%) than acute pain (4%; *p* = 0.024). Participants with chronic pain (mean = 0.7, SD = 1.1) also reported a significantly greater number of visits to the ED in the past five years due to pain compared to participants with acute pain (mean = 0.2, SD = 0.6; *p* = 0.042).

Logistic regression assessed the association of various factors on the likelihood that participants would use a prescription medication to manage their pain. The final model was statistically significant (*p* < 0.001) and contained three independent variables: type of pain (acute or chronic), number of visits to the ED because of pain in the past five years, and impact of pain on their ability to perform daily activities.

## 4. Discussion

This is the first study to report the prevalence of pain, as well as self-reported use of pain management strategies and pain outcomes, among the student pharmacist population. The two most important findings from this study were that 15% of the student pharmacists in this study have had chronic pain in the past five years that impacted their ability to attend school and work. Nearly all students surveyed used a combination of medical and non-medical strategies to manage their pain resulting in the use of a large number of strategies, which indicates that pain management can create a substantial burden on students while they attend school. A third important finding is the greater use of prescription medications by students with chronic pain, compared to those with acute pain, and that most differences between men and women were related to use of opioids, OTC NSAIDs, and non-medical strategies.

### 4.1. Chronic Pain Among Student Pharmacists

A significant portion of student pharmacists in this study (15%) reported experiencing chronic pain in the past five years, which is higher than the national level of eight percent reported in Nahin’s 2012 National Institute of Health (NIH) study, although Nahin’s study used a one-year timeframe [4]. However, our study finding is supported by Mallen and colleagues’ study from England that found 14% of 18–25-year-old adults sampled had chronic pain [9]. Participants of the current study with chronic pain reported an impact on their daily activities significantly more than participants with acute pain (*p* = 0.024). Furthermore, students with chronic pain reported that pain impacted their ability to work (15%) and their ability to attend school (9%), ‘quite a bit’ or ‘very much’. Intuitively, adequate pain management is required in order to perform activities of daily living [10] and attend work or school, yet studies have found that the effect of chronic pain on work performance has been underestimated since many people continue to work with pain [11,12]. A study by Stewart and colleagues’ using the Work and Health Interview found 13% of the workforce was less productive due to pain [12]. The current study findings should prompt educational institutions and others concerned with the health of higher education and health professions students, an imminent workforce, to assess the prevalence and impact of pain. To combat the adverse effects of pain and maintain productivity, provision of academic and self-care support for students suffering from pain should be offered to help ensure that their full educational potential is met.

Student pharmacists reported the most common location of pain was the head. Data from the National Centers for Health Statistics indicated that headache or migraine pain was common (15% of adults) and that young adults aged 18–44 were almost three times as likely to report head pain compared to older adults 65 years and older [13]. Our study findings are consistent with that data. Headaches and migraines may be more common in the younger student population due to fatigue, stress of studying, reading small-print texts, and excessive use of technology [14], compared to the general population. Again, educational institutions may educate students on the importance of self-care including proper sleep, diet, exercise and limiting screen time to ease fatigue, reduce stress, and improve pain, especially headaches.

### 4.2. Student Pharmacists’ Pain Management Strategies

Student pharmacists with chronic pain reported significantly greater use of a range of pain management strategies which is expected as individuals with chronic pain are more likely to seek medical attention and prescriptive interventions to manage their pain [15]. Student pharmacists also commonly used a large number and variety of non-medical strategies, which may seem counterintuitive given their advanced pharmacological knowledge. Many of the non-medical strategies employed by participants have been found to help manage pain in previous studies [16,17,18]. For example, superficial heat can help reduce pain and enable patients with chronic pain to begin an appropriate exercise regime that may help manage pain [19]. Reasons why student pharmacists commonly chose to use non-medical strategies were not investigated, but this finding raises important questions about how student pharmacists’ education and knowledge impacts their choice of pain-management strategy. Perhaps, due to greater awareness and knowledge about potential side effects of the medications, student pharmacists seek non-medical strategies to manage their pain or at least employ medical and non-medial strategies in combination. The majority (96%) of participants in this study used both medical and non-medical strategies, which supports the notion that this combined approach is most appropriate for chronic pain management [18,19].

The literature suggests that there are differences in pain perception between men and women: Keogh and Denford found differences in pain between sexes [20], and Tsang et al. found that chronic pain was more prevalent among women than men [5]. In our study, no significant difference was found between men and women, although there was a trend toward women having more acute and chronic pain (*p* = 0.066). Both men and women used NSAIDs most commonly to help manage their pain; prescription NSAIDs were used similarly by both genders, but women used OTC NSAIDs more often than men. The cause of pain was not investigated in this survey, but it may be that women were using more NSAIDs to manage dysmenorrhea (menstrual pain), prevalent in this age group [21]. Given that NSAIDs are considered one of the first-line medical strategies for pain [22] and are easily obtained by OTC purchase, their frequent use could be expected. This finding is also supported by Hirsh and colleagues’ study that found clinicians recommended OTC medications and heat or cold strategies most frequently, but opioids least frequently [23]. Notably, in the current study, men reported using opioids (a prescription-only product in the US) more often than women, which contradicts the previous findings of a report that found opioid use was more prevalent among women [24]. The reason for this difference is unclear, but it could be due to small sample size or differences in the age of the sample and requires further investigation. We also found that women used non-medical strategies such as rest, relaxation or stress reduction techniques, and changing body position more often than men. These findings have important implications for pharmacists, where an opportunity exists to intervene and educate the large number of people seeking OTC strategies, such as NSAIDs, for pain management. Appropriate use of medical and non-medical pain management strategies should be promoted to improve health and quality of life of patients.

### 4.3. Implications for Pharmacy Education

The findings from this study have implications for colleges of pharmacy. The study indicated that, at any point in time, around 15% (including students with acute and chronic pain) of students are experiencing pain while they are attending school. An estimate of 15% translates to 60 students in a professional program with 100 students per class or 120 students in a college with 200 students per class—a meaningful number of students. Pain creates a substantial burden for students; they must obtain the medical care, manage treatment strategies, and deal with how the pain and its treatment interfere with their academics [25,26]. Approximately one-tenth of students experienced side effects from their pain management strategies, which creates an additional burden for the students. Moreover, more than a third of students with pain stated that they were not satisfied with their current pain management strategies. These observations indicate that, to assure student success, it is important for colleges to have clearly stated policies and procedures for both students and faculty for dealing with pain or other burdensome illness.

Future research may seek to determine a more precise estimate of the burden of illness on student pharmacists from pain, and to investigate the effectiveness of institutional policies and support on academic success of the students. Furthermore, it would be informative to understand how the pain management strategies used in this sample of student pharmacists compare to non-healthcare students, other healthcare students, or practicing healthcare professionals. Logistic regression indicated that participants who had more frequently visited the ED were more likely to have used prescription drugs to manage their pain; future research could be conducted with a larger sample to identify health resource utilizations and additional factors that affect health and social outcomes of student populations.

Limitations of this study included those inherent to survey research, such as assumptions about participants understanding the questions (including knowledge of drug classes) and answering them accurately. This survey was designed to assess the type of pain most bothersome to respondents, thus was unable to capture any incidences of co-occurring acute and chronic pain. The findings also were likely affected by the response rate although a 70% response rate is considered adequate. The small sample size of student pharmacists was not demographically representative of the US population and therefore not generalizable outside of the study population.

## 5. Conclusions

In this sample of student pharmacists, 15% reported having chronic pain in the past five years, which impacted their ability to attend school and work. The student pharmacists surveyed did not rely solely on medications and medical strategies for managing pain; rather they used a combination of medical and non-medical strategies. There was some difference between students with chronic pain and acute pain, primarily related to the use of prescription medications. In addition, the primary difference between men and women was that women were more likely to use non-medical strategies. Educational institutions should ensure they have clear policies and procedures for students and faculty when dealing with pain or other illness to promote academic success. Future research is needed to explore strategies to better support students with chronic pain during their education, including investigating the precise burden, and establishing and measuring the effectiveness of policies and support systems.

## Figures and Tables

**Table 1:** Demographic and pain characteristics of study participants.

	Acute	Chronic	No Pain	*p* Value
Total Number (*n* = 218)	140 (64)	33 (15)	45 (21)	
Age *n* (%)				0.897
19–25	97 (69)	23 (70)	33 (73)	
26+	42 (30)	10 (30)	12 (27)	
Sex *n* (%)				0.066
Male	50 (36)	14 (42)	25 (56)	
Female	89 (64)	19 (58)	20 (44)	
Year in program *n* (%)				0.490
First year	46 (33)	15 (45)	18 (40)	
Second year	52 (37)	12 (36)	13 (29)	
Third year	41 (29)	6 (18)	14 (31)	
Pain intensity mean (SD) ^1^	5.2 (2.0)	5.9 (1.7)	-	0.035 ^2^
Level of pain tolerated mean (SD) ^1^	6.1 (1.9)	6.3 (1.8)	-	0.544

Numbers may not equal 100% due to rounding and missing data; *n* = number of participants; SD = standard deviation; ^1^ Rating scale from 0 (no pain) to 10 (worst pain imaginable); ^2^ Significant at *p* < 0.05.

**Table 2:** Medical and non-medical strategies used by student pharmacists for the self-management of pain.

	Acute *n* (%)	Chronic *n* (%)	*p* Value	Male *n* (%)	Female *n* (%)	*p* Value
Total Number	140 (81)	33 (19)		64 (37)	108 (63)	
Medical strategies requiring a prescription						
NSAIDs	47 (34)	19 (58)	0.011 ^1^	25 (39)	41 (38)	0.866
Other	32 (23)	13 (39)	1.00	22 (34)	23 (21)	1.000
Opioids	20 (14)	3 (9)	0.574	13 (20)	10 (9)	0.040 ^1^
Muscle relaxants	7 (5)	12 (36)	<0.001 ^1^	8 (13)	11 (10)	0.640
Physical therapy	6 (4)	9 (27)	<0.001 ^1^	9 (14)	6 (6)	0.056
Surgery	7 (5)	4 (12)	0.224	4 (6)	6 (6)	1.000
Steroid injections	4 (3)	4 (12)	0.044 ^1^	4 (6)	4 (4)	0.472
TENS	3 (2)	4 (12)	0.026 ^1^	2 (3)	5 (5)	1.000
Over the counter strategies						
NSAIDs	106 (76)	29 (88)	0.129	45 (70)	90 (83)	0.045 ^1^
Acetaminophen	76 (54)	21 (64)	0.330	31 (48)	66 (61)	0.105
Aspirin	15 (11)	2 (6)	0.533	6 (9)	11 (10)	0.863
Herbal/dietary supplements	4 (3)	4 (12)	0.044 ^1^	3 (5)	5 (5)	1.000
Other	5 (4)	1 (3)	1.000	2 (3)	4 (4)	1.000
Non-medical strategies						
Rest	97 (69)	22 (67)	0.770	38 (59)	81 (75)	0.032 ^1^
Relaxation/stress reduction	55 (39)	18 (55)	0.110	20 (31)	53 (49)	0.022 ^1^
Hot/cold packs	53 (38)	18 (55)	0.080	26 (41)	45 (42)	0.893
Changing body position	48 (34)	17 (52)	0.066	18 (28)	47 (44)	0.044 ^1^
Hot baths/showers	49 (35)	16 (48)	0.150	20 (31)	45 (42)	0.173
Massage	42 (30)	14 (42)	0.170	21 (33)	35 (32)	0.956
Avoid specific activities	35 (25)	15 (45)	0.020 ^1^	19 (30)	31 (29)	0.891
Physical activity	28 (20)	15 (45)	0.002 ^1^	17 (27)	26 (24)	0.716
Other	18 (13)	10 (30)	0.682	9 (14)	19 (18)	1.000
Meditation	22 (16)	5 (15)	0.936	10 (16)	17 (16)	0.984

Numbers may not equal 100% due to rounding and missing data; only students with acute or chronic pain are included in this table as they are the population of interest; *n* = number of participants; NSAIDs = non-steroidal anti-inflammatory drugs; TENS = transcutaneous electrical nerve stimulator; ^1^ Significant at *p* < 0.05.

**Table 3:** Student pharmacist reported outcomes of pain self-management strategies.

	Acute	Chronic	*p* Value
Total Number (*n* = 173)	140 (81)	33 (19)	
Satisfaction with current pain management strategies *n* (%)			0.889
Not/Somewhat/Moderately	51 (36)	13 (39)	
Satisfied	54 (39)	12 (36)	
Very	35 (25)	7 (21)	
Side effects experienced with current pain management *n* (%)			0.111
Yes	11 (8)	6 (18)	
No	113 (81)	25 (76)	
Overall health status *n* (%)			0.096
Poor/Fair	11 (8)	5 (15)	
Good/Excellent	129 (92)	28 (85)	
Effect of pain on ability to perform daily activities *n* (%)			0.024 ^1^
Somewhat or less	133 (95)	28 (85)	
Quite a bit/very much	5 (4)	5 (15)	
Effect of pain on ability to participate in leisure activities *n* (%)			0.249
Somewhat or less	130 (93)	29 (88)	
Quite a bit/very much	8 (6)	4 (12)	
Effect of pain on ability to work *n* (%)			0.173
Somewhat or less	127 (91)	28 (85)	
Quite a bit/very much	10 (7)	5 (15)	
Effect of pain on ability to attend school *n* (%)			0.446
Somewhat or less	130 (93)	30 (91)	
Quite a bit/very much	8 (6)	3 (9)	
Effect of pain on relationships with other people *n* (%)			0.693
Somewhat or less	128 (91)	32 (97)	
Quite a bit/very much	10 (7)	1 (3)	
Level of pain when pain strategies employed mean (SD) ^2^	3.0 (2.2)	3.2 (2.4)	0.641
Emergency department visits in last 5 years due to pain mean (SD)	0.2 (0.6)	0.7 (1.1)	0.042 ^1^

Numbers may not equal 100% due to rounding and missing data; only students with acute or chronic pain are included in this table as they are the population of interest; *n* = number of participants; SD = standard deviation; ^1^ Significant at *p* < 0.05; ^2^ Rating scale from 0 (no pain) to 10 (worst pain imaginable).

## Supplementary Materials

The following are available online at www.mdpi.com/2226-4787/6/1/11/s1, Appendix S1: Questionnaire.
