# Supplementary material for: An Exploratory Study of Student Pharmacists’ Self-Reported Pain, Management Strategies, Outcomes, and Implications for Pharmacy Education
Source: Pharmacy (Basel). 2018 Jan 22;6(1):11. doi: 10.3390/pharmacy6010011 (PMC5874550; doi:10.3390/pharmacy6010011)
Supplement: Supplementary file 1 [file pharmacy-06-00011-s001.pdf]

Supplementary Materials

Please answer the questions as accurately as you can.

Section 1: This section asks questions about the last time you experienced pain, the intensity of the pain, and where the pain occurs. Circle the one best response for each question.

1. When was the last time that you experienced pain so intense that you felt that you needed to do something to control it?

(a)Today
(b)Within the past week
(c)Within the past month
(d) Within the past 3 months
(e) Within the past 6 months
(f) Within the past year
(g) Within the past 5 years
(h) I don't remember when I experienced pain so intense that I felt that I needed to do something to control it.

(If you answered (h), please proceed to question #20.)

2. How would you describe the pain that is most bothersome to you? Select the one statement that most accurately describes your most bothersome pain.

(a) Acute—you experience pain only occasionally, most of the time you are pain free
(b) Chronic—you have had pain almost every day for 3 of the past 6 months
(c) Chronic-intermittent—you have reoccurring pain mixed with pain free intervals

3. Thinking back to your most recent episode of pain, indicate on the scale below the intensity of the pain.

0 1 2 3 4 5 6 7 8 9 10
No pain at all Worst pain imaginable

4. On the scale below, indicate the level at which you can tolerate pain.

0 1 2 3 4 5 6 7 8 9 10
No pain at all Worst pain imaginable

5. Thinking back to your most recent episode of pain, where is (was) your pain primarily located?

(a) Head
(b) Neck/shoulders
(c) Arms/hands
(d) Chest
(e) Abdomen
(f) Back
(g) Legs/feet

Section 2: This section of the questionnaire asks about what you do to manage your pain, the effectiveness of the management strategies that you use, and whether you experience any side effects from any of the treatments.

6. Which of the following therapies for pain management that **require a prescription**, do you use to manage pain? Circle all that apply.

(a) NSAIDs
(b) Anti-epileptic medications
(c) Beta blockers/calcium channel blockers
(d) Tricyclic antidepressants/SSRIs/SNRIs
(e) Barbiturates/butalbitals
(f) Muscle relaxants
(g) Narcotic analgesics
(h) Triptans
(i) Physical therapy
(j) TENS
(k) Surgery
(l) Ergotamines
Other________________________________ (m) Steroid injections

7. Which of the following OTC medications do you use to manage pain? Circle all that apply.

(a) ASA
(b) Acetaminophen
(c) Other____________________________________
(d) NSAIDS
(e) Herbal remedies/dietary supplements

8. Which non-medical strategies do you use to manage pain? Circle all that apply.

(a) Changing body position
(b) Relaxation/stress reduction
(c) Meditation
(d) Avoid specific activities
(e) Hot baths/showers
(f) Physical activity
(g) Other_____________________________________
(h) Hot/cold packs
(i) Massage
(j) Acupuncture
(k) Chiropractor
(l) Rest
(m) Education

9. Thinking about all of the above strategies that you use for managing pain, including those that require a prescription, OTC medications, and non-medical strategies, what are the 3 most effective strategies for managing your pain?

(a) The most effective strategy is: __________________________________
(b) The second most effective strategy is: ___________________________
(c) The third most effective strategy is: _____________________________

10. Considering all the pain management strategies that you typically use, what is the level of your pain?

0 1 2 3 4 5 6 7 8 9 10

No pain at all Worst pain imaginable

11. How satisfied are you with your current pain management strategies (circle one answer please)?

(a) Not at all satisfied
(b) Somewhat satisfied
(c) Moderately satisfied
(d) Satisfied
(e) Very satisfied

12. Have you ever experienced any side effects from any of the treatments that you use?

No Yes
If yes, with which treatments?

Treatment (example: NSAIDS) **Side effect of treatment**

____________________________ ____________________________
____________________________ ____________________________
____________________________ ____________________________

Section 3: This section of the questionnaire is about the outcomes of your pain management strategies, specifically whether pain interferes with different aspects of daily living and how you would rate your overall health.

13. How many times have you visited the emergency department in the last 5 years because of your pain?

Number of times = __________

14. Overall, how would you rate your health?

(a) Poor
(b) Fair
(c) Good
(d) Excellent

This question asks you how much your pain interferes with different types of activities. Select the one best response for each activity.

How much does pain interfere with your:

	Not at all	A little bit	Somewhat	Quite a bit	Very much
15. Daily activities?	☐	☐	☐	☐	☐
16. Ability to participate in leisure activities?	☐	☐	☐	☐	☐
17. Ability to work?	☐	☐	☐	☐	☐
18. Ability to attend school?	☐	☐	☐	☐	☐
19. Relationships with other people?	☐	☐	☐	☐	☐

Section 4: This section asks some basic demographic questions about you.

20. What year of the professional pharmacy program are you in?

(a) 1st year
(b) 2nd year
(c) 3rd year
(c) 4th year

21. What is your gender?

(a) Male
(b) Female

22. What is your age?

(a) ≤18
(b) 19–25
(c) 26–30
9d) 31–35
(e) 36–40
(f) ≥41

23. What is your marital status?

(a) Married
(b) Divorced
(c) Separated
(d) Never married
(e) Living with partner

24. In the space below, please describe any issues related to the self-management of pain that you think are important.
